# Measuring visually guided motor performance in ultra low vision using virtual reality

**DOI:** 10.3389/fnins.2023.1251935

**Published:** 2023-12-20

**Authors:** Arathy Kartha, Roksana Sadeghi, Chris Bradley, Brittnee Livingston, Chau Tran, Will Gee, Gislin Dagnelie

**Affiliations:** ^1^Wilmer Eye Institute, Johns Hopkins School of Medicine, Baltimore, MD, United States; ^2^Department of Biological and Vision Sciences, State University of New York College of Optometry, New York, NY, United States; ^3^Herbert Wertheim School of Optometry and Vision Science, University of California, Berkeley, Berkeley, CA, United States; ^4^Central Association for the Blind and Visually Impaired, Utica, NY, United States; ^5^BMORE VIRTUAL LLC, Baltimore, MD, United States

**Keywords:** hand-eye coordination, virtual reality, ultra low vision, outcome measures and assessments, vision restoration

## Abstract

**Introduction:**

Ultra low vision (ULV) refers to profound visual impairment where an individual cannot read even the top line of letters on an ETDRS chart from a distance of 0.5 m. There are limited tools available to assess visual ability in ULV. The aim of this study was to develop and calibrate a new performance test, Wilmer VRH, to assess hand-eye coordination in individuals with ULV.

**Methods:**

A set of 55 activities was developed for presentation in a virtual reality (VR) headset. Activities were grouped into 2-step and 5-step items. Participants performed a range of tasks involving reaching and grasping, stacking, sorting, pointing, throwing, and cutting. Data were collected from 20 healthy volunteers under normal vision (NV) and simulated ULV (sULV) conditions, and from 33 participants with ULV. Data were analyzed using the method of successive dichotomizations (MSD), a polytomous Rasch model, to estimate item (difficulty) and person (ability) measures. MSD was applied separately to 2-step and 5-step performance data, then merged to a single equal interval scale.

**Results:**

The mean ± SD of completion rates were 98.6 ± 1.8%, 78.2 ± 12.5% and 61.1 ± 34.2% for NV, sULV and ULV, respectively. Item measures ranged from −1.09 to 5.7 logits and − 4.3 to 4.08 logits and person measures ranged from −0.03 to 4.2 logits and −3.5 to 5.2 logits in sULV and ULV groups, respectively. Ninety percent of item infits were within the desired range of [0.5,1.5], and 97% of person infits were within that range. Together with item and person reliabilities of 0.94 and 0.91 respectively, this demonstrates unidimensionality of Wilmer VRH. A Person Item map showed that the items were well-targeted to the sample of individuals with ULV in the study.

**Discussion:**

We present the development of a calibrated set of activities in VR that can be used to assess hand-eye coordination in individuals with ULV. This helps bridge a gap in the field by providing a validated outcome measure that can be used in vision restoration trials that recruit people with ULV, and to assess rehabilitation outcomes in people with ULV.

## Introduction

Ultra-low vision (ULV) refers to a level of vision where a person lacks form vision, but is able to differentiate light and dark, and can see moving shadows and/or silhouettes (Geruschat et al., 2012; Nau et al., 2013). In the clinic, people with ULV would not be able to see the top line of letters on an ETDRS chart even from a distance of 0.5 m, equivalent to 20/1600 (Endo et al., 2016). Currently, visual acuity in individuals with ULV can only be measured using specially designed tests such as the Berkeley Rudimentary Vision Test (BRVT) (Bailey et al., 2012), Freiberg Visual Acuity Test (FrACT) or Basic Assessment of Light and Motion (BALM) Test (Bach et al., 2010). Although these tests can estimate acuities as low as 20/63000, it is not clear what level of visual task performance can be expected in people with ULV. So, the question is, what kind of visually guided activities of daily living (ADL) can be performed using ULV?

The ultra-low vision visual functioning questionnaire (ULV-VFQ) (Jeter et al., 2017) was designed to assess self-reported difficulty in performing activities of daily living in individuals with ULV. Most of the activities in the ULV-VFQ that are related to visual information gathering under both low contrast and lighting conditions were rated as most difficult by participants with ULV, followed by visuomotor, mobility and shape discrimination activities while using ULV. However, there has been a lack of tests comparing these self-reports with actual task performance. Recently, we reported on the design and development of an assessment in virtual reality (Wilmer VRI) that can be used to measure visual information gathering in individuals with ULV (Jeter et al., 2017).

Using the Wilmer VRI, we showed that visual information gathering in ULV varies with ambient lighting and contrast which was consistent with findings for the ULV-VFQ. We demonstrated that under low visual demands (high contrast, no clutter), people with ULV are able to perform a range of visual information gathering tasks such as deciding whether the room lights are ON or OFF, a computer screen is ON or OFF, or a candle is lit in a dark room (Kartha et al., 2023); locating a missing white plate on a black table or a white tube of cream on a black countertop. However, their performance on other tasks such as identifying the orientation of white window blinds (horizontal/vertical) on a brightly lit window, locating a white pill on a white countertop, deciding whether a candle is lit in a bright room were near or at chance levels. Thus, we showed that individuals with ULV were able to perform spatial localization and discrimination tasks with the least difficulty under high visibility conditions and low cognitive demands.

It is important to broaden the range of performance measures beyond the domain of visual information gathering, because many daily activities reported by individuals with ULV involve other functional domains, in particular hand-eye coordination, and wayfinding. Visual ability in these domains should be assessed since clinical trials for vision restoration recruit people with ULV or restore vision to ULV levels (Geruschat et al., 2012; Nau et al., 2013). Therefore, in the present study, we extend the assessment of visual performance by individuals with ULV to visuomotor tasks, again in virtual reality.

Previous studies have reported the kinematics of reach and grasp movements in individuals with low vision and blindness. Pardhan et al. (2011) reported an increase in onset time, total time, time to maximum velocity and time after maximum grip aperture that were more pronounced in people with a more recent onset of central vision loss compared to those who had vision loss for more than 10 years. In another study, Gonzalez-Alvarez et al. (2007) measured 3D kinematics of reaching and grasping movements and reported decreased maximum velocity and increased maximum grip aperture among normally sighted individuals with artificially restricted field of view. Cavallo et al. (2017) found that previous visual experience and visual input had little effect on performing reach and grasp movements with different anticipatory end goals (e.g., grasp to pass, grasp to pour, grasp to place) when comparing normally sighted volunteers with and without visual input and early and late blind individuals.

These were studies in the real-world where the participants were provided haptic feedback. We on the other hand are interested in studying performance of real-world activities by individuals with ULV using visual cues only. In the real-world, haptic feedback cannot be eliminated and therefore, performance can be heavily dependent on haptic cues, especially for people with profoundly impaired vision. This made it preferable to present our activities in VR where we could emulate real-world daily activities while providing only visual information.

We are interested in developing outcome measures that are meaningful in real life, and more informative for both clinicians and patients than customary visual function outcomes. Currently, a two- or three-line improvement in ETDRS visual acuity is considered clinically significant for most vision restoration trials, and thus the criterion for a successful intervention by the clinicians. However, this may not translate to any measurable difference in real-world tasks, so patients may not consider their treatment to be effective, leading to disappointment, frustration and poor compliance to rehabilitation and training. Conversely, failure to reach the pre-set change in visual acuity may ignore meaningful improvements in everyday activities perceived by the patients, and these may be captured by calibrated performance measures. This is all the more important because the precision of visual acuity tests such as the FrACT and BRVT is not as good as that of ETDRS visual acuity.

The aim of this study, therefore, was to develop a validated and calibrated performance assessment (Wilmer VRH) in the visuomotor domain to assess hand-eye coordination for individuals with ULV that can be used as an outcome measure in clinical trials. As mentioned, we chose to implement the assessment in virtual reality, so that it can be used across multiple centers and populations with uniform settings across visits and participants by presenting only visual cues. The test was designed specifically as a toolkit for testing in ULV, however we collected normative data from healthy young adults under two visibility levels to evaluate if the activities designed and the set up in virtual reality allow completion by typical participants without extensive training and are sensitive to differences in visual ability levels.

## Methods

### Study design

In this prospective study, we present the design and development of the Wilmer VRH, a test to assess visuomotor performance using hand-eye coordination tasks in ULV. As there have been no previous studies that assessed hand-eye coordination in ULV using virtual reality, we first tested normally-sighted participants who performed the test with their native vision and also under simulated ULV (sULV) conditions (Dagnelie et al., 2021; Singh et al., 2022) (details under ‘*Participants*’). This was done to validate our approach in VR and also to evaluate content validity for testing in ULV. All scenes in VR were presented in an HTC VIVE Pro Eye headset with a screen resolution of 1440×1600 pixels per eye, diagonal field of view of 110 degrees and a frame rate of 90 Hz. Following this, we tested participants with actual ULV and performed Rasch analysis to calibrate the items.

### Virtual environment

All activities were presented in immersive VR, that is, head position and orientation are used to adjust the scene representation, and it is indistinguishable from reality, regardless of eye position; eye-tracking had no effect on the scene. Users could freely scan the scene around them. All participants were using their native vision, whether normal, sULV, or ULV.

### Test description

We identified a total of 55 activities (items) from an inventory of daily activities developed from previous focus group discussions in individuals with ULV (Adeyemo et al., 2017), so that the items chosen were relevant for the ULV population. For example, daily meal preparation, playing games and personal healthcare are a few areas of interest reported by individuals with ULV. Using these as criteria, we designed the activities in Wilmer VRH after discussion with clinicians, occupational therapists, and O&M specialists. For each activity, we identified distinct steps that need to be completed for the activity to be successful. For example, cooking a pancake involves locating the bottle with pancake batter, pouring the batter onto a pan, locating the spatula, flipping the pancake using the spatula and transferring the pancake onto a plate. A full list of activities and steps is provided in Supplementary Table 1. See Supplementary videos for sample videos of participant performance.

### Hand tracking

**Figure 1:**
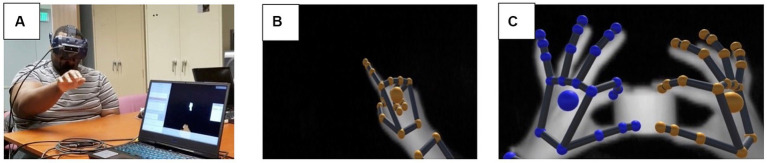
**(A)** A subject performing the test with the headset and the LMC attached to the headset; **(B)** a grayscale image capture of right hand from the LMC’s sensors; **(C)** grayscale image of the hands holding an object in 3D space and the color-coded visualization based on raw camera images of finger positions. The images in panels **(B,C)** were captured using the LMC hand tracking software that is part of the LeapMotion package.

A Leap Motion Controller (LMC) (UltraLeap™) (Figure 1) was attached to the headset to track hand movements in 3D space. The system captures gray scale stereo images of objects directly illuminated by infrared LEDs on the LMC and reconstructs a 3D representation of the hands using proprietary algorithms.

### Item structure

**Figure 2:**
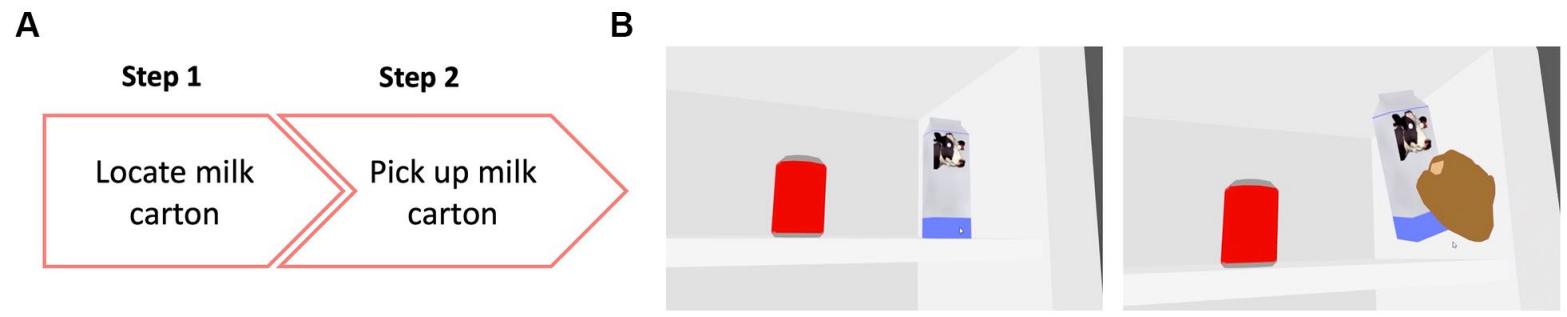
**(A)** Example of a 2-step item where the participant was asked to locate and pick up a milk carton from inside a refrigerator. **(B)** View inside the VR environment showing the participant performing the task. On the left panel is the view of the scene with the milk carton and a soda can, which was a distractor object. On the right panel is the subject locating and correctly picking up the milk carton.

**Figure 3:**
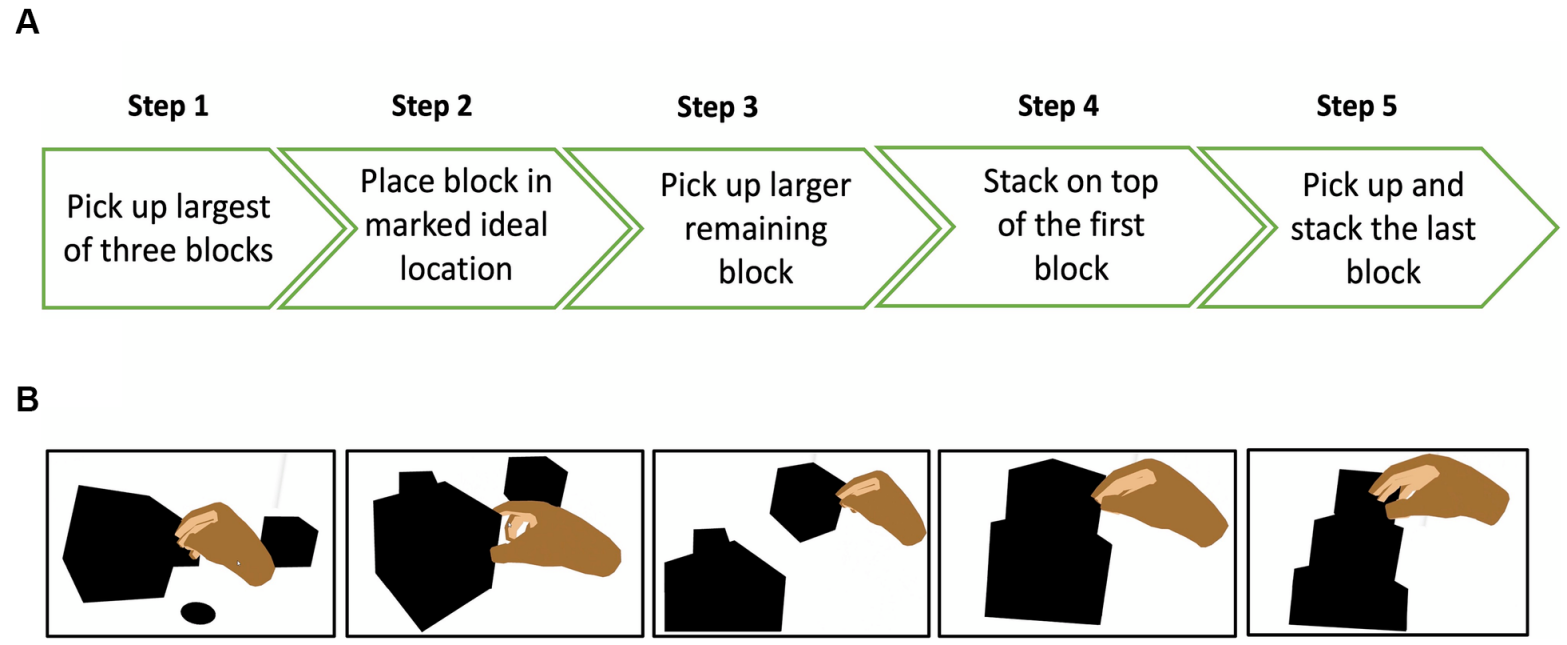
**(A)** Example of a 5-step item where the participant was asked to make a tower by stacking 3 blocks of different sizes, starting with the biggest block **(B)** view of the different steps in the VR environment.

We designed each task (item) such that it had 2 or 5 steps, and a participant was allowed to move to step n + 1 only after completing step n. This allowed us to give credit for each step successfully completed. 33 items had 2 steps (Figure 2) while 22 had 5 steps (Figure 3). All tasks were designed so that it was possible for the VR system to automatically determine whether the step was completed. For example, in the stacking the block task, the system detects when the hand contacts the first block and extracts the time that each event occurred. Participants performed a range of tasks including reaching and grasping, stacking, throwing, cutting, sorting, and pointing. All items were presented at three visibility levels (high, medium, low) by varying the contrast, size, speed, clutter, or distance to the target, with the exception of three items that involved objects with different contrast levels and sizes within them and had repetitive steps — two of these items (baking cookies, cooking a pancake) were only presented at one visibility level, while the third one (place settings on a dining table) was presented at two visibility levels.

All participants were given the opportunity to practice the tasks prior to the actual test. During the practice trials participants were given auditory feedback when they made errors (a beep when dropping or misplacing an item) as well as when they performed correctly (a ‘ding’ sound of a bell). Test trials and practice trials were not identical. The practice trials were to familiarize participants with the activity in the VR environment, so they were able to manipulate objects without haptic feedback. No verbal feedback was provided during any of the test trials. The order of items was randomized between participants.

For the normally sighted group, the order of testing with normal vision and simulated ULV was randomized between participants. The examiner controlled the testing via a laptop and started the test by clicking a ‘start’ button on the user interface. The examiner had the option to advance or go back to the previous item as well as to repeat the item as needed. At the end of each task, the examiner had the option to enter comments or make remarks about each item. Participants’ hand movements were tracked by the software and each step completed was recorded along with the time stamp. The set-up time was approximately 5 min, and testing was completed within 2 h.

### Scoring

A score of ‘1’ was given for each step successfully completed and a score of ‘0’ otherwise. For those items where distances were measured from a target (e.g., throwing a ball into a basket) and touch time (touching a moving ball), the measurements were divided into quintiles so that it fits into a 5-step format and then scored. The quintile scores ranged from 1 to 5 with values in the first quintile given a score of ‘1’.

### Data analysis

As described, we had two sets of activities in Wilmer VRH – a set of activities with 2-steps and a set with 5-steps. No assumptions were made about the relative difficulties of 2-step and 5-step items. Our goal was to estimate item and person measures for these activities on a common scale. Typically, the Partial Credit Model (PCM) would be used to analyze this type of data, where the number of steps (i.e., number of thresholds) is not the same for all items. However, the PCM estimates disordered thresholds in many cases, and this has been demonstrated to be a result of the mathematics of the PCM not being consistent with the assumption that a rating scale is always defined by ordered thresholds (Bradley and Massof, 2018). This led us to use the method of successive dichotomizations (MSD), which is a polytomous Rasch model that always estimates ordered thresholds. The problem with MSD is that it assumes the same number of rating categories for all items. This means that we had to estimate item and person measures using MSD separately for all 2-step items and for all 5-step items, and then combine the item scales in a second step.

It is important to note that MSD estimates parameters in the same measurement units for any number of rating categories — which means that it is not *a priori* a problem that 2-step and 5-step items have different numbers of rating categories — but with different origins. By convention, the origin of the Rasch logit scale is the mean item measure, which can be interpreted as the mean difficulty for all items, and there is no *a priori* reason why the mean difficulty of all 2-step items should be the same as the mean difficulty for all 5-step items.

To place all 2-step and 5-step item measures on the same scale, we must estimate the offset between the origins of the 2-step and 5-step scales. This was done by first estimating the item and person measures for 2-step and 5-step items separately. Then we anchored (i.e., fixed) the 5-step person measures to re-estimate the 2-step item measures and vice versa. This allowed us to estimate the (average) offset between the mean difficulties of the 2- and 5-step items, and then shift all 5-step item measures onto the 2-step item measure scale. Finally, we anchored the combined set of item measures and re-estimated all person measures.

### Participants

We recruited 20 normally sighted volunteers who completed testing under normal vision and simulated ULV conditions. ULV was simulated using a combination of Bangerter filters that blurred visual acuity to approximately 20/2000 (2.0 logMAR). We then collected data from 33 participants with ULV at the Ultra-Low Vision lab at Johns Hopkins (*n* = 14) and Central Association for the Blind and Visually Impaired, Utica, NY (*n* = 19). All participants signed an informed consent before participating in the study; the study protocol was approved by the Johns Hopkins Medicine IRB and adhered to the tenets of the Declaration of Helsinki.

## Results

Among the ULV participants, 20 were females. The age range was from 29 to 93 years, with a mean age of 59.7 years. The main causes of ULV were: AMD (21.2%), glaucoma (18.2%), diabetic retinopathy (12.1%), retinitis pigmentosa (12.1%), optic atrophy (9.1%), cone-rod dystrophy (6.1%), retinal detachment (3%), retinopathy of prematurity (3%), corneal opacity (3%) and idiopathic intracranial hypertension (3%). Among healthy volunteers, 12 were females. Their age range was 20–66 years with a mean age of 36 years.

### Completion rates

**Figure 4:**
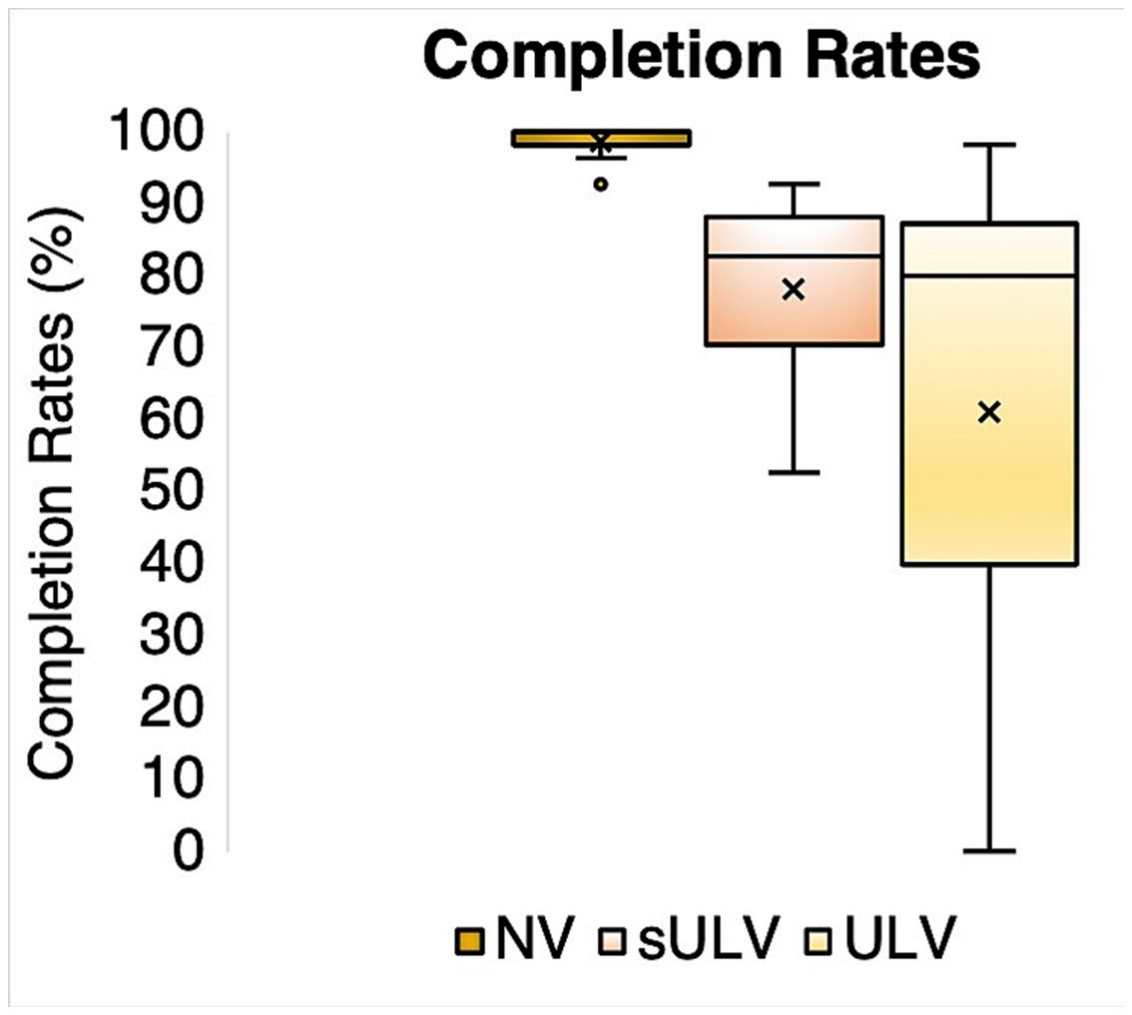
Completion rates for normal vision (NV), simulated ULV (sULV) and ULV participants.

To examine the validity of testing performance in VR and the test’s content validity, we evaluated whether our normally sighted participants could complete all tests under normal vision and simulated ULV conditions. Figure 4 shows the completion rates for the normal vision (NV), simulated ULV (sULV), and ULV groups. The mean ± SD of overall completion rates were 98.6 ± 1.8%, 78.2 ± 12.5% and 61.1 ± 34.2% for the NV, sULV, and ULV groups, respectively. Since nearly all participants in our NV group were able to complete nearly all the steps in the activities — causing a ceiling effect — the MSD analysis would not be able to assign person measures, and further results will be presented for sULV and ULV only.

### MSD analysis

#### Item measures

The estimated offset between item measures for 2-step and 5-step was 0.8 logit for ULV and 1.0 logit for sULV. The final estimated item measures after adjusting for the offsets ranged from −1.09 to 5.7 logits for sULV and −4.3 to 4.08 logits for ULV. We note that participants in the sULV group could not perform the first step for 15 activities so, item measures could not be estimated for these activities.

**Figure 5:**
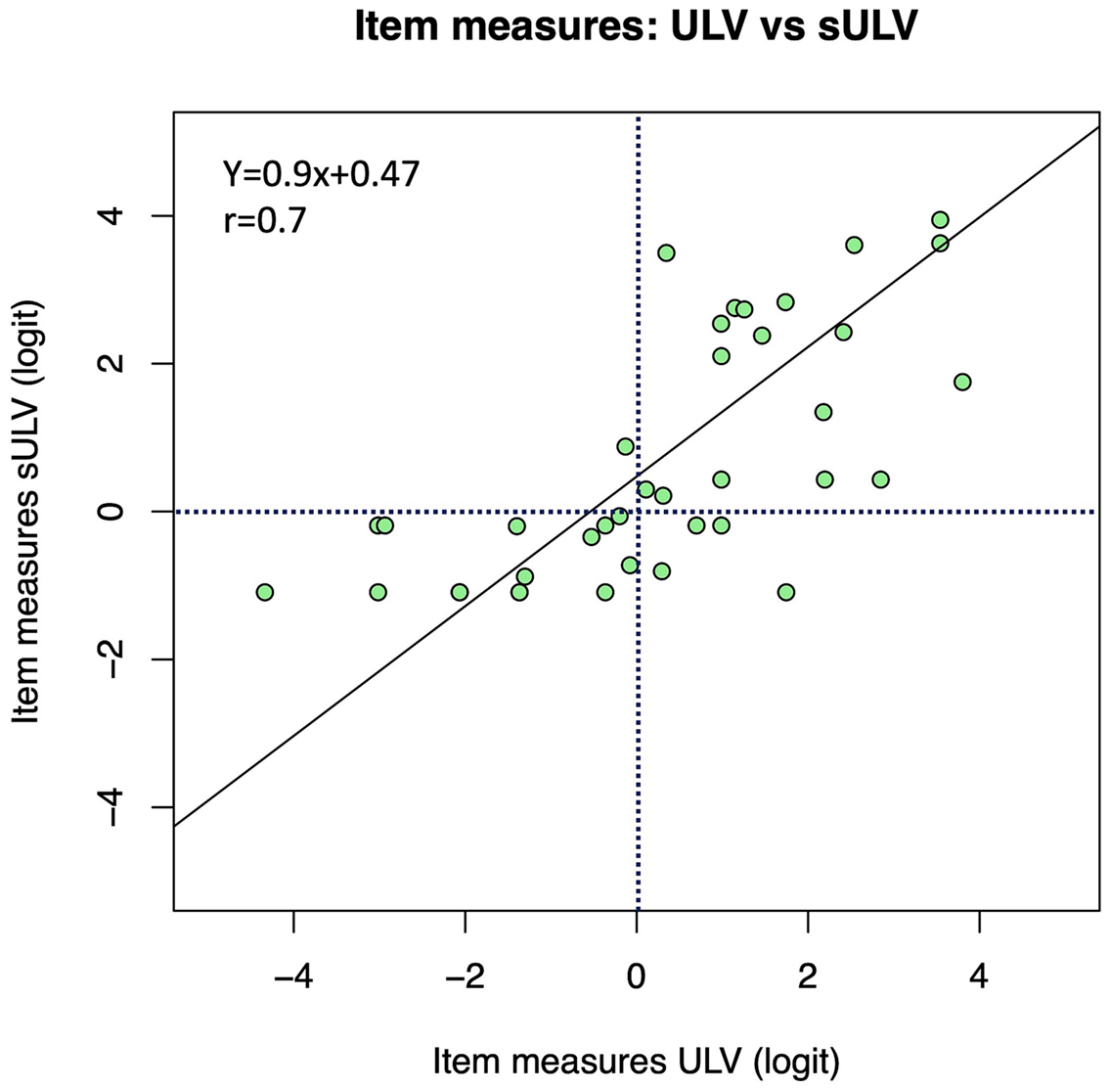
Relationship between item measures for sULV and ULV. Overall, the item measures were more positive for the sULV group.

Figure 5 shows the relationship between item measures for sULV and ULV, with a correlation coefficient *r* = 0.7. The slope of the Deming regression line was 0.9, which shows that item measures scale similarly in sULV and ULV. This indicates that visually guided performance was comparable between sULV and ULV groups, despite the small offset for individual items. The Y- intercept gives the best estimate of the offset between the item measures for sULV and ULV. This offset is caused by the fact that, by convention, the mean of all item measures in a given sample is zero, but the two scales will differ for different samples. Since sULV and ULV consist of two entirely different samples, the average difficulty estimates for the two samples show an offset of, in this case 0.47 logit. If the two samples were identically distributed, the offset between the scales would be zero and the slope would be 1. Our results show that item measures for the two samples scale very similarly.

**Figure 6:**
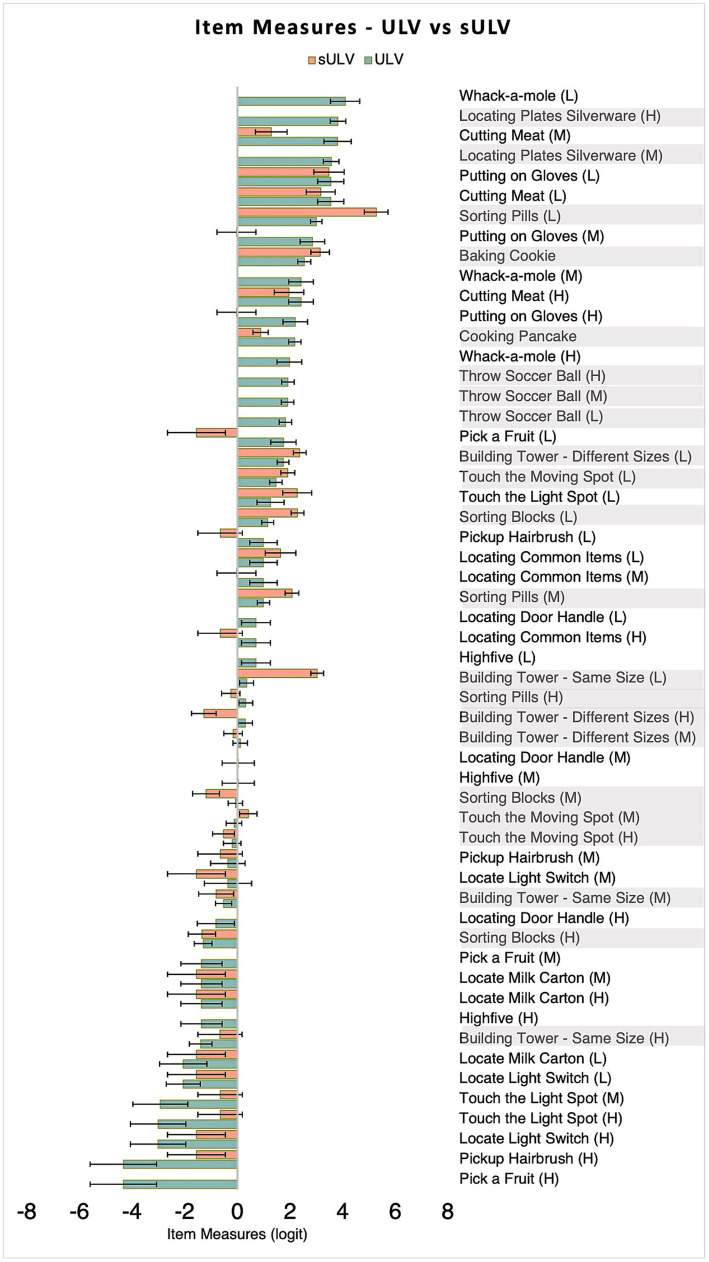
Item measures for sULV and ULV groups. Item measures for sULV have been adjusted for the offset between the two sets so that sULV and ULV are on the same scale. A more positive item measure indicates a more difficult item. The lighter bars indicate 2-Step activities and darker bars indicate 5-Step activities. Error bars indicate standard errors. For most activities, sULV yielded a more positive item measure compared to ULV. H, M and L stand for high, medium and low visibility levels.

Figure 6 shows individual item measures estimated for each activity ranked from the most difficult (towards the top) to the least difficult (towards the bottom). As shown, 27 (49%) of the activities were easier for people with ULV compared to those with sULV, likely due to their long-term adaptation to vision loss compared to sULV which was transient, induced by Bangerter foils. This gives task-wise comparisons between item measures for sULV and ULV groups. It has to be noted that some item measures were not estimated when subjects could not complete any of the steps and therefore were considered as missing data in the analysis.

#### Person measures

**Figure 7:**
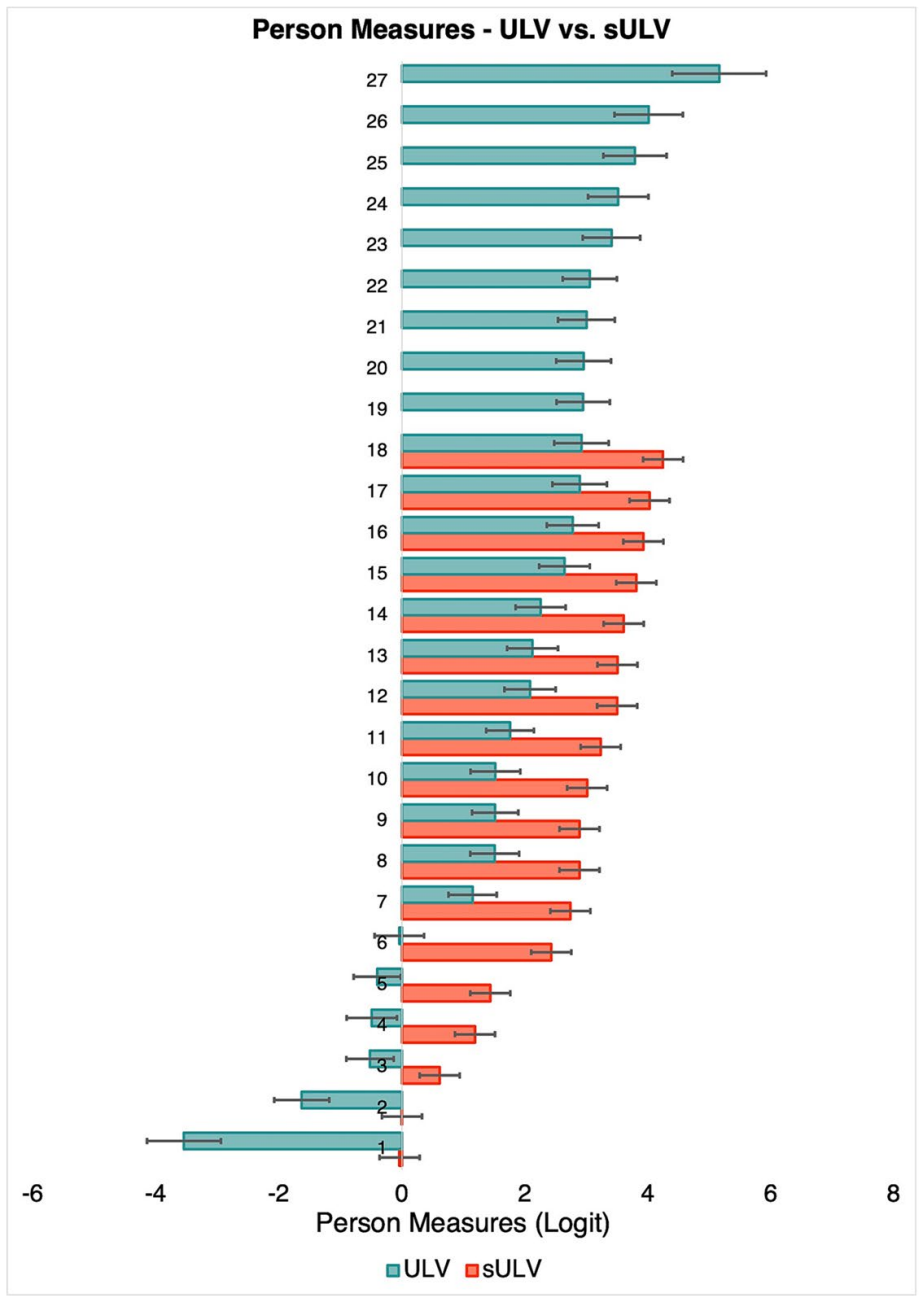
Person measures for individual participants. Error bars indicate standard errors.

Figure 7 shows estimated person measures for sULV and ULV. Person measures ranged from −0.03 to 4.2 logit and −3.5 to 5.2 logit for the sULV and ULV groups, respectively. The mean ± SD was 2.6 ± 1.4 logit and 1.9 ± 1.9 logit for sULV and ULV groups, respectively. The range of estimated person measures for the sULV group was narrower than for the ULV group; this makes sense given that all sULV participants were wearing the same filter that reduced the vision to similar levels across participants, as opposed to the heterogeneous group of individuals with ULV. There were two participants in the sULV group and 5 participants in the ULV group who could not complete any of the activities and we therefore could not estimate their person measures.

### Targeting and Unidimensionality

**Figure 8:**
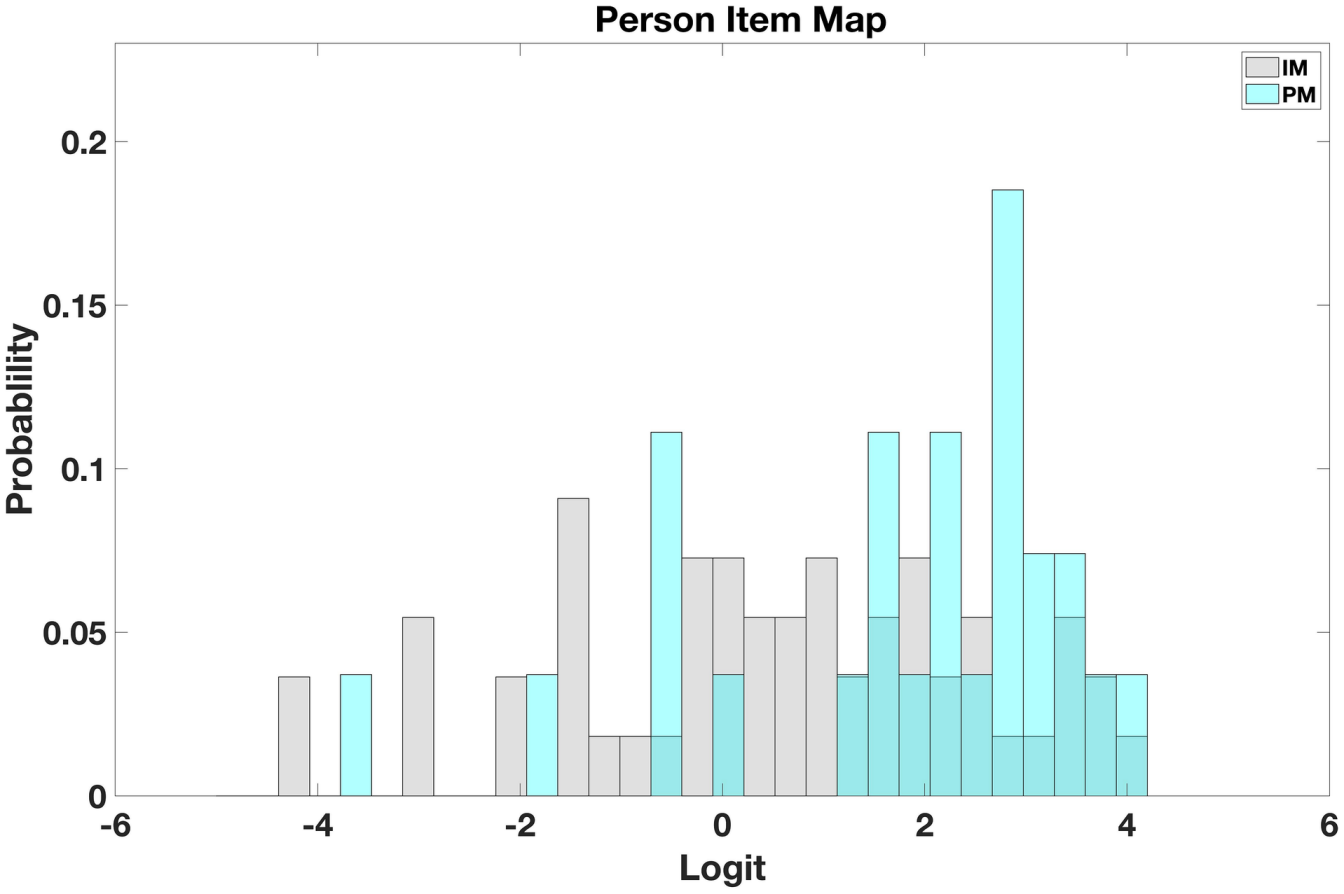
Person Item map for ULV. The map shows that persons and item measures are similarly distributed, demonstrating that the instrument is well-targeted for the ULV population.

Figure 8 shows histograms of person and item measure distributions for the ULV group. The two distributions cover a similar range, showing that the instrument is well-targeted for individuals with ULV.

Unidimensionality is a desired property of measurement instruments. In Rasch analysis it can be evaluated through the infit mean square statistic, or infit. Approximately 90% of the estimated item infits and 94% of person infits were in the desired range of [ 0.5,1.5] (Gagnier et al., 2021). This together with item and person reliabilities of 0.94 and 0.91, respectively, shows the unidimensionality of the instrument.

## Discussion

In the present study, we developed and calibrated a set of activities to assess hand-eye coordination in individuals with ULV using virtual reality. Calibration means that we have estimated a set of item measures from the target population that can be used in other studies to estimate person measures on the same scale (i.e., other studies do not need to estimate their own item measures). The activities were based on the ULV inventory, to make sure they are relevant for a ULV population. The calibrated Wilmer VRH fills a gap in the field of ULV by providing an outcome measure for visual performance in clinical trials. Unlike questionnaires where the responses are subjective, our outcomes reflect task performance closer to real life scenarios. Even though we used VR, the perfect scores from normally sighted participants demonstrate that VR was not a limiting factor in completing the activities.

The main outcome measures of the study are item and person measures that represent item difficulty and person ability. A novelty of our approach is that we were able to combine two different types of items, 2-step, and 5-step, along a common scale. This was made possible by using MSD, which yields item measure estimates in the same units regardless of the number of steps in each item. After combining the two sets of item measures on a common scale by removing the offset, we can compare persons against items — more able persons and more difficult items towards one end of the scale and less able persons and less difficult items towards the other end.

Item measures and person measures can be used to compare performance within participants over a period, e.g., to monitor changes in performance with disease progression, or to compare performance pre and post intervention, e.g., before and after treatment or rehabilitation. One can expect that the participants will be able to move up the scale from less difficult items towards more difficult ones, corresponding to the positive shift in their person measures, as a sign of effective treatment or rehabilitation. A shift exceeding the 95% confidence interval, i.e., 1.96 times the standard error of their person measure estimate, would represent a statistically significant change. Since such a change in performance measures would also reflect changes in their activities of daily living, this would also represent a clinically significant change.

The use of VR in our assessment, while lacking the contribution of haptic, and possibly auditory, cues offered by real world activities, provides a convenient way to study visual performance in isolation. When performing real-world activities, individuals with ULV, much more so than better sighted individuals, make highly effective use of non-visual cues, so studying visual performance in isolation in a ULV population greatly benefits from the use of VR. Note that, in terms of visual information provided, VR and real-world activities are indistinguishable to someone with ULV, since they cannot see the subtle differences between the two that a better sighted person might notice.

An important difference between our study and previous studies (Gonzalez-Alvarez et al., 2007; Pardhan et al., 2011, 2017; Cavallo et al., 2017; Gerb et al., 2022) is the range of activities we chose to use in this study. Real-world activities are rarely limited to reach and grasp but involve detailed interactions with the objects and the environment. By choosing a range of activities, i.e., some involve pointing, some involve stacking, some purely based on reaction time (playing whack-a-mole) and some others requiring distance and depth judgments (throwing a ball into a basket), we have attempted to capture the breadth of activities involved in real-world performance. To streamline the analysis of such a diverse set of activities we used a multi-step MSD analysis with binary scoring; this was the most effective way to arrive at an assessment that would yield a person ability measure along an equal interval scale. In the future, further refinements can be explored by extracting kinematics in this assessment and compare them with those in real-world activities.

The results from our study are not limited to a particular type of vision loss (e.g., central vs. peripheral). The ULV cohort in the present study came with a diverse group of eye diseases, representative of the ULV population. We found that the difficulty of items for those with sULV was higher than for those with ULV. Nevertheless, item measures for the sULV and ULV groups were linearly related with a slope of 0.9, which supports content validity of this instrument, regardless of the cause of vision loss. All participants with sULV were nominally given the same level of visual impairment. Not surprisingly, then, person measures were more widely spread out for the ULV group whose visual impairment levels varied widely, from finger counting to bare light perception. The wide range of person measures demonstrates the ability of the test to differentiate among members of the ULV group. Incidentally, members of the ULV group are more adapted to their vision loss compared to sULV who had short-term visual loss, so it stands to reason that the spread in person measures among the ULV group is primarily due to their vision level, whereas for the sULV group it is due to differences in ability to adapt to sudden deterioration.

There are a few limitations to this study. First, our sample size was not large enough to provide a fully calibrated set of item measures; this can be addressed by follow-up studies in additional ULV cohorts. Second, we could not study ULV populations based on long-term vs. short-term differences in adaptation to vision loss, since most of our participants with ULV had long-term visual impairment and therefore, could have developed strategies to perform some of these tasks non-visually. Our sULV group mimics the effect of short-term vision loss; however: They were given the same visual restrictions, and thus approximate the impact of short-term vision loss. Finally, we did not include tactile and auditory information in VR. However, this was intentional, as mentioned, since we wanted to estimate visual ability in isolation.

This study covers the second of three visual domains that can be used to categorize activities in ULV: visual information gathering, hand-eye coordination and wayfinding. Once the wayfinding instrument (Wilmer VRW) is completed, these three sets of activities will constitute a full set of validated outcomes for use in clinical trials enrolling patients with ULV.

## Data availability statement

The raw data supporting the conclusions of this article will be made available by the authors, without undue reservation.

## Ethics statement

The studies involving humans were approved by Johns Hopkins Institutional Review Board. The studies were conducted in accordance with the local legislation and institutional requirements. The participants provided their written informed consent to participate in this study.

## Author contributions

AK: design, data collection, analysis, and writing. RS: design, data collection, and writing. CB: development of MSD and data analysis. BL: data collection. CT: unity programming. WG: software development and consultation. GD: conceptualization and design and Supervision.

## Supplementary material

SUPPLEMENTARY VIDEO 1 A participant performing the Whack-a-Mole task (left) and the view of the monitor from the examiner’s side (right).

SUPPLEMENTARY VIDEO 2 Performance of “Locate a Milk Carton” Task.

SUPPLEMENTARY VIDEO 3 Performance of “Locate Common Items” Task – participants are asked to pick up a wallet, phone and scissors in the correct order from among a group of objects on the table.

SUPPLEMENTARY VIDEO 4 A participant performing “stacking blocks” task.
