# Supplementary material for: Measuring visually guided motor performance in ultra low vision using virtual reality
Source: Front Neurosci. 2023 Dec 20;17:1251935. doi: 10.3389/fnins.2023.1251935 (PMC10765526; doi:10.3389/fnins.2023.1251935)
Supplement: Supplementary file 5 [file Data_Sheet_1.PDF]

No.	Item	Task Description	Visibility	Steps
	**5-step Tasks**			
1	Cooking Pancakes	Pour batter on to a pan to make a pancake, flip the pancake with a spatula and then transfer to a plate	High	Pour batter into pan
				Scoop Up Pancake
				Flip back into pan
				Sccop up again
				Place on plate
2	Baking Cookies	Scoop the cookie batter from a bowl and tap it on the marked area on the cookie sheet	High	Measure the distance from the mark on the sheet and divide the distance into quintiles.
3	Locating Plates/Cutlery	Placing a knife, fork, plate, napkin and cup on their designated spots on a placemat	High	Place plate close enough to ideal plate location
				Place fork close enough to ideal fork location and orientation
				Place knife close enough to ideal knife location and orientation
				Place napkin close enough to ideal napkin location and orientation
				Place cup close enough to ideal cup location
4	Locating Plates/Cutlery	Placing a knife, fork, plate, napkin and cup on their designated spots on a placemat	Medium	Place plate close enough to ideal plate location
				Place fork close enough to ideal fork location and orientation
				Place knife close enough to ideal knife location and orientation
				Place napkin close enough to ideal napkin location and orientation
				Place cup close enough to ideal cup location
5	Throw Ball	throw a ball into a basket - 3 trials/short distance	High	Measuring the distance from the basket and estimate quintiles
6	Throw Ball	throw a ball into a basket - 3 trials/medium distance	Medium	Measuring the distance from the basket and estimate quintiles
7	Throw Ball	throw a ball into a basket - 3 trials/far distance	Low	Measuring the distance from the basket and estimate quintiles
8	Sorting Pills	Pick up pills in the order of size starting from the biggest and then putting it into the basket	High	Pick up largest from set of three pills
				Put pill in box
				Pick up larger pill from a set of two pills
				Put pill in the box
				Pick up last pill and put in the box
9	Sorting Pills	Pick up pills in the order of size starting from the biggest and then putting it into the basket	Medium	Pick up largest from set of three pills
				Put pill in box
				Pick up larger pill from a set of two pills
				Put pill in the box
				Pick up last pill and put in the box
10	Sorting Pills	Pick up pills in the order of size starting from the biggest and then putting it into the basket	Low	Pick up largest from set of three pills
				Put pill in box
				Pick up larger pill from a set of two pills
				Put pill in the box
				Pick up last pill and put in the box
11	Tower Build Same Size	To stack blocks one on top of the other to build a tower	High	Pick up the first block
				Place the first block close enough to the ideal location/orientation
				Pick up the second block
				Stack on top of the first block
				Pick up and stack the last block
12	Tower Build Same Size	To stack blocks one on top of the other to build a tower	Medium	Pick up the first block
				Place the first block close enough to the ideal location/orientation
				Pick up the second block
				Stack on top of the first block
				Pick up and stack the last block
13	Tower Build Same Size	To stack blocks one on top of the other to build a tower	Low	Pick up the first block
				Place the first block close enough to the ideal location/orientation
				Pick up the second block
				Stack on top of the first block
				Pick up and stack the last block
14	Tower Build Different Size	To stack the blocks by size to make a tower making sure that the biggest block is the bottom	High	Pick up largest from set of three blocks
				Place the first block close enough to the ideal location/orientation
				Pick up the largest remaining block
				Stack on top of the first block
				Pick up and stack the last block
15	Tower Build Different Size	To stack the blocks by size to make a tower making sure that the biggest block is the bottom	Medium	Pick up largest from set of three blocks
				Place the first block close enough to the ideal location/orientation
				Pick up the largest remaining block
				Stack on top of the first block
				Pick up and stack the last block
16	Tower Build Different Size	To stack the blocks by size to make a tower making sure that the biggest block is the bottom	Low	Pick up largest from set of three blocks
				Place the first block close enough to the ideal location/orientation
				Pick up the largest remaining block
				Stack on top of the first block
				Pick up and stack the last block
17	Sorting Blocks	Pick up blocks in the order of size starting from the biggest and then putting it into the basket	High	Pick up largest from set of three blocks
				Put block in box
				Pick up larger block from a set of two blocks
				Put block in the box
				Pick up last block and put in the box
18	Sorting Blocks	Pick up blocks in the order of size starting from the biggest and then putting it into the basket	Medium	Pick up largest from set of three blocks
				Put block in box
				Pick up larger block from a set of two blocks
				Put block in the box
				Pick up last block and put in the box
19	Sorting Blocks	Pick up blocks in the order of size starting from the biggest and then putting it into the basket	Low	Pick up largest from set of three blocks
				Put block in box
				Pick up larger block from a set of two blocks
				Put block in the box
				Pick up last block and put in the box
20	Touch the Moving Light Spot	Touch the light spot and follow it across the screen without losing track	High	Measure the total touch time and divide into quintiles. The higher the touch time higher the score.
21	Touch the Moving Light Spot	Touch the light spot and follow it across the screen without losing track	Medium	Measure the total touch time and divide into quintiles. The higher the touch time higher the score.
22	Touch the Moving Light Spot	Touch the light spot and follow it across the screen without losing track	Low	Measure the total touch time and divide into quintiles. The higher the touch time higher the score.
	**2-step Tasks**			
23	Locating Door Handle	Grab the door handle and push the door open	High	Grab correct door handle
				Open door by at least 10 degrees
24	Locating Door Handle	Grab the door handle and push the door open	Medium	Grab correct door handle
				Open door by at least 10 degrees
25	Locating Door Handle	Grab the door handle and push the door open	Low	Grab correct door handle
				Open door by at least 10 degrees
26	Pick up Hair Brush	pick up a hairbrush lying on the bathroom sink	High	Locate hairbrush from 4 possible locations
				Grab hairbrush
27	Pick up Hair Brush	pick up a hairbrush lying on the bathroom sink	Medium	Locate hairbrush from 4 possible locations
				Grab hairbrush
28	Pick up Hair Brush	pick up a hairbrush lying on the bathroom sink	Low	Locate hairbrush from 4 possible locations
				Grab hairbrush
29	Touch Light Spot	point to a spot of light located in 4 possible random locations	High	Locate light spot from 4 possible locations
				Point to the light spot
30	Touch Light Spot	point to a spot of light located in 4 possible random locations	Medium	Locate light spot from 4 possible locations
				Point to the light spot
31	Touch Light Spot	point to a spot of light located in 4 possible random locations	Low	Locate light spot from 4 possible locations
				Point to the light spot
32	Put on Gloves	align the correct hand in the correct orientation on the glove and hold for 2 seconds	High	Locate the glove
				Place correct hand in correct orientation
33	Put on Gloves	align the correct hand in the correct orientation on the glove and hold for 2 seconds	Medium	Locate the glove
				Place correct hand in correct orientation
34	Put on Gloves	align the correct hand in the correct orientation on the glove and hold for 2 seconds	Low	Locate the glove
				Place correct hand in correct orientation
35	Locate Light Switch	flip the light switch	High	Locate light switch from 4 possible locations
				flip light switch
36	Locate Light Switch	flip the light switch	Medium	Locate light switch from 4 possible locations
				flip light switch
37	Locate Light Switch	flip the light switch	Low	Locate light switch from 4 possible locations
				flip light switch
38	Locate Milk carton	pick up the milk carton from among other items in the fridge	High	Locate milk Carton among 2 objects
				Pick milk carton
39	Locate Milk carton	pick up the milk carton from among the other items in the fridge	Medium	Locate milk Carton among 3 objects
				Pick milk carton
40	Locate Milk carton	pick up the milk carton from among other items in the fridge	Low	Locate milk Carton among 4 objects
				Pick milk carton
41	Pick Fruit	pick the fruit from the tree	High	Locate Black Grapes from 3 possible locations
				Pick Black Grapes
42	Pick Fruit	pick the fruit from the tree	Medium	Locate Red Grapes from 3 possible locations
				Pick Red Grapes
43	Pick Fruit	pick the fruit from the tree	Low	Locate Green Grapes from 3 possible locations
				Pick Green Grapes
44	Give High Five	give high five to a person using the correct hand	High	Locate the raised hand
				Give high five
45	Give High Five	give high five to a person using the correct hand	Medium	Locate the raised hand
				Give high five
46	Give High Five	give high five to a person using the correct hand	Low	Locate the raised hand
				Give high five
47	Locate common Items	pick up wallet, phone and scissors in the correct order from among other items on the table among 4 objects	High	Locate the object
				Pick up object
48	Locate common Items	pick up wallet, phone and scissors in the correct order from among other items on the table among 5 objects	Medium	Locate the object
				Pick up object
49	Locate common Items	pick up wallet, phone and scissors in the correct order from among other items on the table among 7 objects	Low	Locate the object
				Pick up object
50	Whack-a-Mole	To whack the mole when it pops up from one of the 6 holes	High	whack the mole at least X out of N times
51	Whack-a-Mole	To whack the mole when it pops up from one of the holes	Medium	whack the mole at least X out of N times
52	Whack-a-Mole	To whack the mole when it pops up from one of the holes	Low	whack the mole at least X out of N times
53	Cutting Meat	To cut the meat into half	High	Grab knife
				Cut the meat in 1/2
54	Cutting Meat	To cut the meat into half	Medium	Grab knife
				Cut the meat in 1/2
55	Cutting Meat	To cut the meat into half	Low	Grab knife
				Cut the meat in 1/2
